# Case Report: Neoadjuvant PD-1 Blockade Plus Concurrent Chemoradiotherapy in Unresectable Locally Advanced Gastric Cancer Patients

**DOI:** 10.3389/fonc.2020.554040

**Published:** 2021-02-05

**Authors:** Jia Wei, Xiaofeng Lu, Qin Liu, Lin Li, Song Liu, Fangcen Liu, Yao Fu, Xiangshan Fan, Yang Yang, Chuang Qi, Yangyang Yu, Wenxian Guan, Baorui Liu

**Affiliations:** ^1^ The Comprehensive Cancer Centre of Drum Tower Hospital, Medical School of Nanjing University & Clinical Cancer Institute of Nanjing University, Nanjing, China; ^2^ Department of General Surgery, Drum Tower Hospital, Nanjing, China; ^3^ Department of Pathology, Drum Tower Hospital, Nanjing, China; ^4^ Department of Radiology, Drum Tower Hospital, Nanjing, China; ^5^ The Medical Department, 3D Medicines Inc., Shanghai, China

**Keywords:** chemoradiotherapy, locally advanced gastric cancer, neoadjuvant, PD-1 inhibitor, immune microenvironment

## Abstract

Programmed death 1(PD-1) blockade has shown promising efficacy in advanced gastric cancer. Here, we performed a retrospective analysis of three patients with locally advanced gastric cancer who received adjuvant PD-1 plus chemoradiotherapy as neoadjuvant treatment. Neoadjuvant sintilimab plus concurrent chemoradiotherapy had an acceptable side-effect profile. All three patients underwent surgical gastrectomy after a median of 3.9 months. A major pathological response occurred in two resected tumors and a pathologic complete response was observed in one patient. Our results suggest that PD-1 blockade combined with chemoradiotherapy is a promising strategy as a neoadjuvant therapy in patients with unresectable locally advanced gastric cancer.

## Introduction

Gastric cancer is one of the most common malignant tumors in the digestive system. The incidence of gastric cancer is higher in Asian countries compared with other regions around the world (1). Surgery remains the best therapy for gastric cancer with resectable tumor. But for the potentially resectable and unresectable patients, preoperative chemoradiation is a recommended option (2). Neoadjuvant chemoradiotherapy may improve overall resectability rate and the complete surgical resection rate of the primary tumor. More specifically, neoadjuvant chemoradiotherapy may eliminate latent micrometastases and reduce the risk of post-surgical recurrence. Thus chemoradiotherapy is regarded as one of the standard treatment options for preoperative treatment of locally advanced gastric carcinoma (3).

Immunotherapy drugs like PD-1/L1 inhibitors have shown promising efficacy in advanced gastric cancer as monotherapy or combined with chemotherapy (4, 5). Recently, some studies have reported that patients with solid cancer exhibited promising response rate to neoadjuvant immune checkpoint blockade. A phase I study has shown promising efficacy of neoadjuvant nivolumab in resectable non-small cell lung cancer with major pathological response rate of 45% (6). In addition, neoadjuvant ipilimumab and nivolumab treatment has also shown to be effective in high-risk resectable melanoma with pathologic complete response rate of 45% (7). Though it has been well established in some solid cancer, the neoadjuvant immunotherapy is a newer approach in Asian gastric cancer patients. Importantly, it has been reported that synergistic effects can be observed when anti-PD-1/L1 antibodies are used in combination with chemotherapy and/or radiotherapy (8). Here, we report the case series of three patients with locally advanced gastric cancer who exhibited a major pathological response to neoadjuvant PD-1 blockade combined with chemoradiotherapy.

## Materials and Methods

### Immunohistochemistry (IHC) Staining

Programmed death ligand-1 (PD-L1) staining using Ventana PD-L1 (SP263) kit (Roche, USA) was performed on a BenchMark XT system (Roche, USA), and the specimen was then counterstained with hematoxylin and coverslipped, according to the manufacturer’s instructions.

### Multiplexed IHC (mIHC) Staining

mIHC was used to identify the immune cell subsets in the tumor immune microenvironment. mIHC staining was performed using PANO7-plex IHC kit (Panovue, Beijing, China), according to the manufacturer’s instructions. Different antibodies were used to identify the immune cells, including anti-CD8 (CST70306, USA), anti-CD56 (CST3576, USA), anti-CD68 (BX50031, China), anti-HLA-DR (ab92511, USA), anti-panCK (CST4545, USA), and anti-S100 (ab52642, USA). S100 staining was used to define the invasive margin and tumor parenchyma. The invasive margin is defined as the region centered on the border separating the normal part from the malignant part, with an extent of 1 mm. The tumor parenchyma corresponds to all the tissue inside the invasive margin. The Mantra System (Akoya, USA) was used to scan the stained slides and build image. The inForm software (Akoya, USA) was used for image analysis.

## Case Presentation

We identified three patients with locally advanced gastric cancer in our hospital from July, 2018 to February, 2019, treated with neoadjuvant anti-PD-1 therapy plus chemoradiotherapy. All of the three patients were gastric adenocarcinoma, MSS status, HER2 negative, and EBV negative. The TNM stage for the three patients was cT3/4aN2M0 with hepatogastric lymph node metastasis (Bulky N2) (Borrmann type 4), cT4a/bN3M0 (Borrmann type 3), cT4a/bN3M0 (Borrmann type 3), respectively. The baseline characteristics for the three patients are summarized in **Table 1**.

**Table 1:** Patients characteristics at baseline.

	Patient 1	Patient 2	Patient 3
Sex	Female	Male	Male
Age	64	69	70
Histology	adenocarcinoma	adenocarcinoma	adenocarcinoma
Grade	3	2	3
Stage	cT3/4aN2M0	cT4a/bN3M0	cT4a/bN3M0
Borrmann type	4	3	3
HER-2	negative	negative	negative
MSI status	MSS	MSS	MSS
EBER	negative	negative	negative

**Figure 1:**
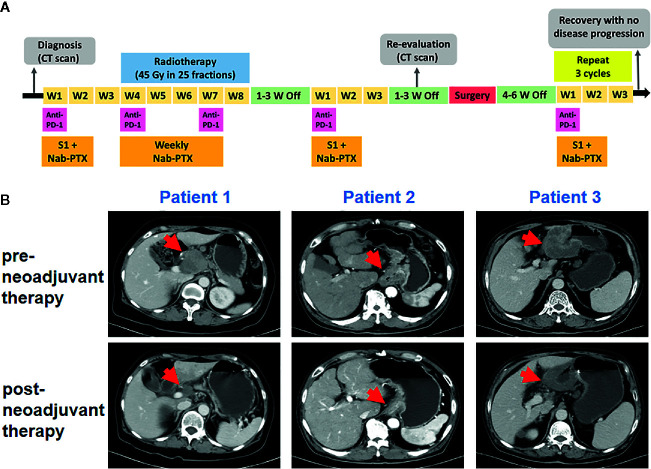
Tumor responses after neoadjuvant therapy. **(A)** Timeline of disease status and corresponding treatment regimens. W, week. **(B)** Tumor lesion of pre- and post-neoadjuvant therapy for three patients.

The three patients were treated with the same therapy. The selected therapeutic regimen consisted of one cycle of PD-1 blockade (sintilimab, 200 mg, on day 1), S-1 (40 mg/m2, twice daily, day 1 to 14), and Nab-PTX (100–120 mg/m2, on day 1 and 8), one cycle of sintilimab (200 mg, every 3 weeks) and weekly Nab-PTX (80–100 mg/m2), in combination with concurrent radiotherapy (RT) (45 Gy in 25 fractions), and another cycle of sintilimab, S-1, and Nab-PTX at the same dose used in the first cycle. After treatment above all, surgery was decided by Multiple Disciplinary Team (MDT). Another three cycles of sintilimab, S-1, and Nab-PTX was used after surgery. The timeline of the clinical treatment was shown in **Figure 1A** (**Figure 1A**). The three patients suffered from neutropenia with grade from 2 to 3. Patient 2 suffered from grade 2 rash and patient 3 suffered from grade 1 anorexia and grade 1 non-immunotherapy related pneumonitis. No deaths or discontinuation due to adverse events occurred. So neoadjuvant sintilimab plus concurrent chemoradiotherapy presented an acceptable side-effect profile.

Pre- and post-neoadjuvant therapy, the computed tomography (CT) was performed in these three patients (**Figure 1B**). For patient 1, the CT had indicated significant reduction of the primary tumor and lymph nodes. For patient 2, tumor and metastatic lymph node volumes were clearly reduced upon CT examination. For patient 3, efficacy evaluation using CT revealed a partial response. All the three patients received surgical D2 gastrectomy. Post-operative pathology showed that a major pathological response occurred in two resected tumors (patient 1 and patient 3) and a pathologic complete response was observed in patient 2. The final TNM stage was ypT3N0M0, ypT0N0M0, and ypT3N2M0, respectively.

**Figure 2:**
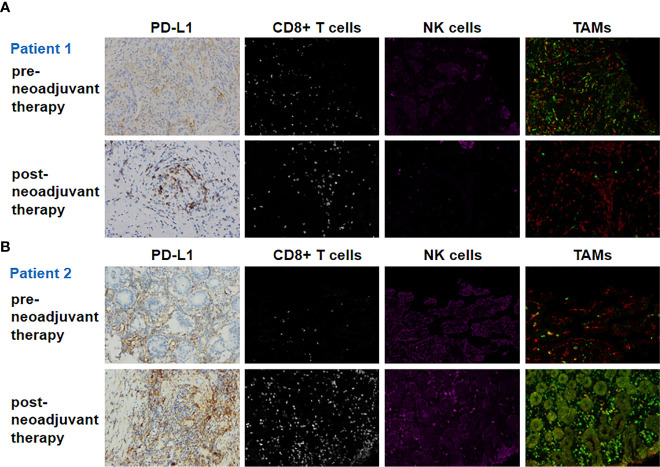
Biomarker findings of the tumor tissue samples. PD-L1, CD8+ T cells, NK cells, and TAMs (tumor-associated macrophages) of pre- and post-neoadjuvant therapy for patient 1 **(A)** and patient 2 **(B)**. Original magnification ×200.

Pre- and post-neoadjuvant therapy immune microenvironment biomarkers were assessed in two patients (patient 1 and patient 2) with adequate tumor tissues (biopsy samples and surgical samples) (**Figure 2**). PD-L1 expression on immune cells (ICs), CD8+ T cells, NK cells, and tumor-associated macrophages (TAMs) were analyzed. For patient 1, pre- and post-neoadjuvant therapy, PD-L1 was expressed on 20% and 10% of ICs. For patient 2, pre- and post-neoadjuvant therapy, PD-L1 was Neoadjuvant therapy of gastric cancer expressed on 40% and 80% of ICs. NK cells were identified using the CD56 marker, and were divided into two categories according to the intensity of membrane staining for the CD56 protein: CD56dim (weak staining) and CD56bright (strong staining). TAMs were identified by CD68 and HLA-DR, and were divided into two categories: type M1 (CD68+ and HLA-DR+) and type M2 (CD68+ and HLA-DR−). These distinctions were made according to the existence of HLA-DR membrane staining. Biomarker analyses are summarized in **Table 2**. For patient 1, biomarker analyses suggested that CD8+ T cells, the ratio of NK bright/NK dim cells and M1/M2 was increased post-neoadjuvant therapy (**Table 2**) both in center and the invasive margin of tumor. Similar results were observed for patient 2 that the increased population of CD8+ T cells, the increased ratio of NK bright/NK dim cells and M1/M2 post-neoadjuvant therapy (**Table 2**). Taken together, these results suggested that the combination of PD-1 blockade and chemoradiotherapy would increase the immune-cell infiltration and the increase of immune-cell infiltration might be associated with the response to PD-1 blockade plus chemoradiotherapy.

**Table 2:** Biomarkers analysis summary.

	Pre-neoadjuvant therapy				Post-neoadjuvant therapy						
Biomarker Types	Solid area		Peripheral area		Solid area			Peripheral area			
	No./mm^2^	%	No./mm^2^	%	No./mm^2^		%	No./mm^2^		%	
**Patient 1**											
CD8	749	2.77	348	0.87	774		5.97	870		3.16	
CD68+HLA-DR+	399	1.48	399	1.00	87		0.67	640		2.32	
CD68+HLA-DR-	1,025	3.79	459	1.15	24		0.19	144		0.52	
CD68+HLA-DR+/CD68+HLA-DR-	0.39		0.87		3.63			4.44			
CD56bright	8	0.03	3	0.01	39		0.3	227		0.82	
CD56dim	667	2.46	56	0.14	66		0.51	90		0.33	
CD56bright/CD56dim	0.01		0.05		0.59			2.52			
**Patient 2**											
CD8	425	1.93	615	2.73	1,180		2.83	2,853		4.62	
CD68+HLA-DR+	73	0.33	73	0.33	1,878		4.51	1,019		1.65	
CD68+HLA-DR-	118	0.53	326	1.45	1,131		2.71	2,597		4.2	
CD68+HLA-DR+/CD68+HLA-DR-	0.62		0.22		1.66			0.39			
CD56bright	21	0.10	9	0.04	795		1.91	230		0.37	
CD56dim	150	0.68	69	0.31	1,604		3.85	642		1.04	
CD56bright/CD56dim	0.14		0.13		0.50			0.36			

T cells are stained for CD8; NK bright cells are stained for CD56bright, NK dim cells are stained for CD56dim; M1 are stained for CD68+HLA-DR+, M2 are stained for CD68+HLA-DR−.

## Discussion

In the present study, we demonstrated three patients with potentially unresectable locally advanced gastric cancer who received neoadjuvant PD-1 blockade plus concurrent chemoradiotherapy. Our results suggest neoadjuvant PD-1 blockade plus concurrent chemoradiotherapy may be efficacious and tolerable in potentially unresectable locally advanced gastric cancer. The increase of immune-cell infiltration may be associated with the response of PD-1 blockade plus concurrent chemoradiotherapy.

Anti-PD-1/PD-L1 therapies have been approved for advanced gastric cancer and further the Checkmate 649 study has shown that Nivolumab plus chemotherapy as first-line treatment for advanced gastric cancer can prolong the overall survival when compared with chemotherapy (5). Meanwhile, anti-PD-1/PD-L1 therapies have shown promising clinical benefit in non-metastasis stages of several cancers (9). The Checkmate 577 study showed that Nivolumab can be used as adjuvant therapy in resected esophageal or gastroesophageal junction cancer (GEJ) (10). For the unresectable non-small-cell lung cancer, the PACIFIC study showed that concurrent chemoradiotherapy followed by anti-PD-L1 drugs prolonged the overall survival when compared with placebo (11). One phase I/II study evaluated the efficacy of avelumab in combination with chemoradiation treatment in stage II/III resectable esophageal and GEJ cancer (12). The chemotherapy drugs and radiation kill tumor cells and promote the release of tumor antigens. This approach may result in a synergistic effect with immuno-checkpoint inhibitors in various therapies targeting malignancies (13). In the cases presented here, the clinical stages of patients were either cT3-4N2 or cT4N3. Regardless, one patient had a pCR and the remaining two patients had MPRs. Even though the final TNM stage for patient 3 was T3N2M0, only a little number of tumor cells was observed in primary tumor and lymph nodes (**Supplemental Figures 1A, B**). Besides, the follow-up had been lasting and until the case series were written, all the three patients were disease free. These results show the promising efficacy of neoadjuvant PD-1 blockade plus concurrent chemoradiotherapy in potentially unresectable locally advanced gastric cancer patients.

The tumor immune microenvironment could reflect the immune response, and changes in the numbers of CD8+ T cells, cancer-associated macrophages, and NK cells infiltrating in the tumor immune microenvironment correlate with clinical outcomes in various malignancies, including gastric cancer, melanoma, lung cancer, and others (14). And studies had also showed that PD-L1 expression and tumor-infiltrating lymphocytes were correlated with pCR in neoadjuvant therapy (15, 16). We found a significant elevation of CD8+ T cells and PD-L1 positivity of ICs after neoadjuvant therapy in patient 2, who achieved pCR after treatment. Several studies have suggested that the density of CD8+ T cells within tumor was higher in PD-1 inhibitor responders than non-responders. Moreover, the CD8+ T cell density in responders was increased during the treatment (17–19). In line with our findings, some studies have indicated that the pathologic and clinical responses of neoadjuvant PD-1 blockade therapy were associated with accumulation of exhausted CD8+ T cells (20, 21). Besides, we also observed the increase of M1/M2 macrophages ratio in patient 1 and 2. M1 macrophages were considered to be activated to participate in anti-tumor immune responses (22). And the ratio of M1/M2 macrophages in an ipilimumab response group have been shown to be significantly higher than that in a non-response group (23).

Our findings could provide a reference for the use of neoadjuvant PD-1 blockade plus concurrent chemoradiotherapy in patients with potentially unresectable locally advanced gastric cancer patients. However, there are some limitations such as the lack of control cases (neoadjuvant treatment with only chemoradiotherapy). In the future, prospective clinical studies with larger samples are required to validate the clinical activity of PD-1 blockade plus concurrent chemoradiotherapy in gastric cancer.

## Ethics Statement

This case series was approved by the local ethical committee. Written informed consent was obtained from the individual(s) for the publication of any potentially identifiable images or data included in this article.

## Author Contributions

JW, WG, and BL conceived the experiments. JW performed most of the experiments. XL, QL, LL, SL, FL, YF, XF, YYA, CQ, and YYU helped with experiments. JW, WG, and BL analyzed the data and wrote the paper. All authors contributed to the article and approved the submitted version.

## Funding

This work was funded by grants from the National Major Projects for “Major New Drugs Innovation and Development”(No.2019ZX09301-150) and Fund for Distinguished Young Scholars of Jiangsu Province (BK20190001).

## Conflict of Interest

Authors CQ and YYY were employed by the company 3D Medicines Inc.

The remaining authors declare that the research was conducted in the absence of any commercial or financial relationships that could be construed as a potential conflict of interest.

## Supplementary Material

Supplementary Figure 1 Hematoxylin and eosin (H&E) staining of patient 3. **(A)** Primary gastric lesions; **(B)** Tumor cells in mucus pool.
