# Supplementary figures and images for: Case Report: Neoadjuvant PD-1 Blockade Plus Concurrent Chemoradiotherapy in Unresectable Locally Advanced Gastric Cancer Patients
Source: Front Oncol. 2021 Feb 5;10:554040. doi: 10.3389/fonc.2020.554040 (PMC7901487; doi:10.3389/fonc.2020.554040)

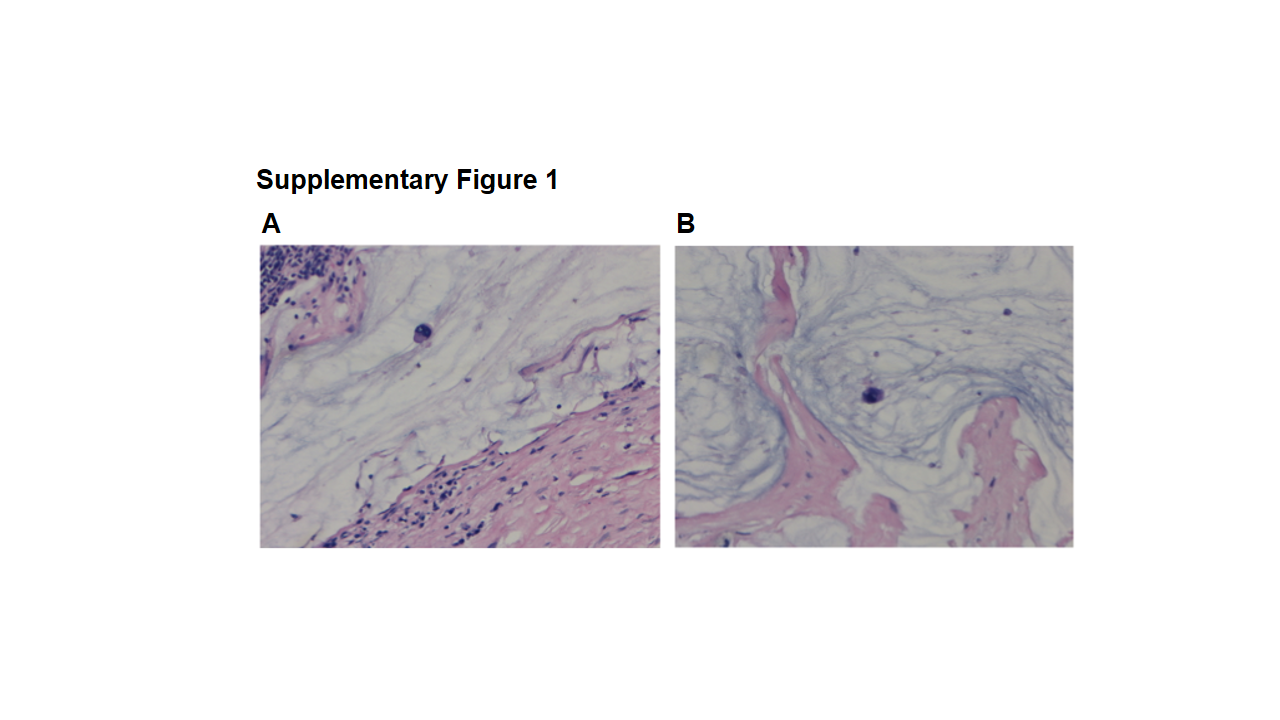

Supplement: Supplementary Figure 1 — Hematoxylin and eosin (H&E) staining of patient 3. (A) Primary gastric lesions; (B) Tumor cells in mucus pool. [file Image_1.tif]
